# Invasive inflammatory fibroid polyp of the stomach: a case report and literature review

**DOI:** 10.1186/s12876-018-0808-9

**Published:** 2018-05-31

**Authors:** Hirofumi Harima, Tokuhiro Kimura, Kouichi Hamabe, Fusako Hisano, Yuko Matsuzaki, Kazutoshi Sanuki, Tadahiko Itoh, Kohsuke Tada, Isao Sakaida

**Affiliations:** 1 Department of Gastroenterology, Ube Industries Central Hospital, 750 Nishikiwa, Ube, Yamaguchi 755-0042 Japan; 2 Department of Pathology, Yamaguchi University Graduate School of Medicine, 1-1-1 Minami-Kogushi, Ube, Yamaguchi 755-8505 Japan; 3 Department of Cancer Screening Center, Ube Industries Central Hospital, 750 Nishikiwa, Ube, Yamaguchi 755-0042 Japan; 4 Department of Surgery, Ube Industries Central Hospital, 750 Nishikiwa, Ube, Yamaguchi 755-0042 Japan; 5 Department of Gastroenterology and Hepatology, Yamaguchi University Graduate School of Medicine, 1-1-1 Minami-Kogushi, Ube, Yamaguchi 755-8505 Japan

**Keywords:** Inflammatory fibroid polyp, Stomach, Invasion, Platelet-derived growth factor receptor alpha mutation

## Abstract

**Background:**

Inflammatory fibroid polyps (IFPs) are rare mesenchymal lesions that affect the gastrointestinal tract. IFPs are generally considered benign, noninvasive lesions; however, we report a case of an invasive gastric IFP. To the best of our knowledge, this is only the second case report of an invasive gastric IFP.

**Case presentation:**

A 62-year-old woman presented with complaints of epigastric pain and vomiting. Computed tomography showed a 27-mm, hyper-enhancing tumor in the prepyloric antrum. Upper endoscopy also showed a submucosal tumor causing subtotal obstruction of the gastric outlet. Because a gastrointestinal stromal tumor was suspected, distal gastrectomy was performed. Histopathological examination revealed spindle cell proliferation in the submucosal layer. The spindle cells had invaded the muscularis propria layer and extended to the subserosal layer. The tumor was finally diagnosed as an IFP based on immunohistochemical findings. No mutations were identified in the platelet-derived growth factor receptor alpha (PDGFRA) gene via molecular genetic analysis.

**Discussion and conclusions:**

After the discovery that IFPs often harbor PDGFRA mutations, these growths have been considered neoplastic lesions rather than reactive lesions. Based on the present case, IFPs might be considered not only neoplastic but also potentially invasive lesions.

## Background

Inflammatory fibroid polyps (IFPs) are rare mesenchymal lesions that affect the gastrointestinal tract. Histologically, spindle cells proliferate in the submucosal layer, and they rarely invade the muscularis propria [1, 2]. For a long time, IFPs were considered reactive lesions [3]; however, recent reports revealed that these growths often contain mutations in the platelet-derived growth factor receptor alpha (PDGFRA) gene [4, 5]. PDGFRA mutations are oncogenic mutations that are often found in gastrointestinal stromal tumors (GISTs) [6]. Recently, IFPs have been considered neoplastic lesions that are not invasive [5]. In the current report, we describe a case of an invasive gastric IFP, and based on this case, we suggest that the behavior of IFPs be reconsidered.

## Case presentation

**Fig. 1:**
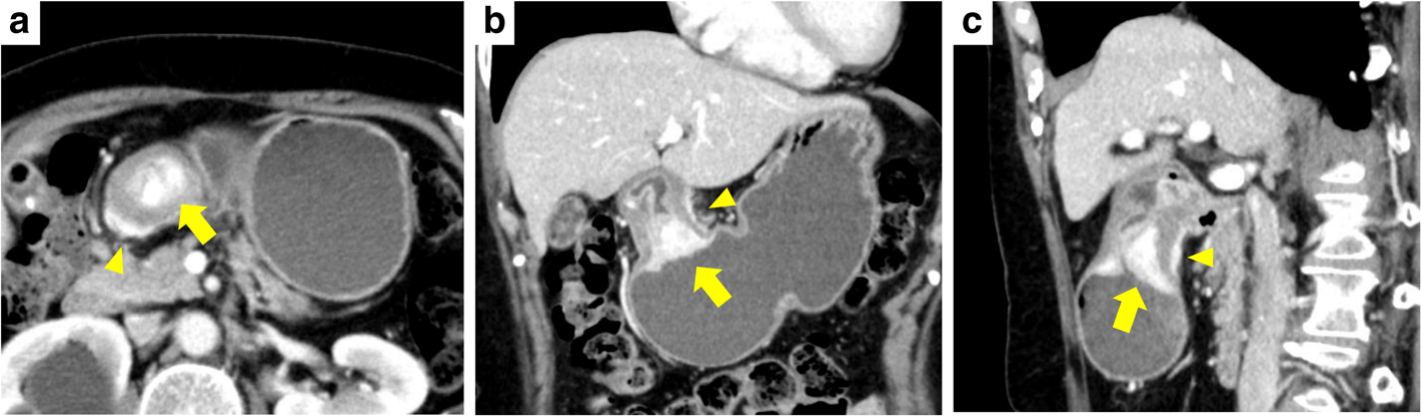
Computed tomography (CT) findings. **a** Axial view. **b** Coronal view. **c** Sagittal view. CT image showing a hyper-enhancing tumor in the submucosa of the prepyloric antrum (*arrows*). CT showing a thickening of the muscularis propria layer and a hyper-enhancing lesion in the subserosa of the gastric antrum (*arrow heads*)

**Fig. 2:**
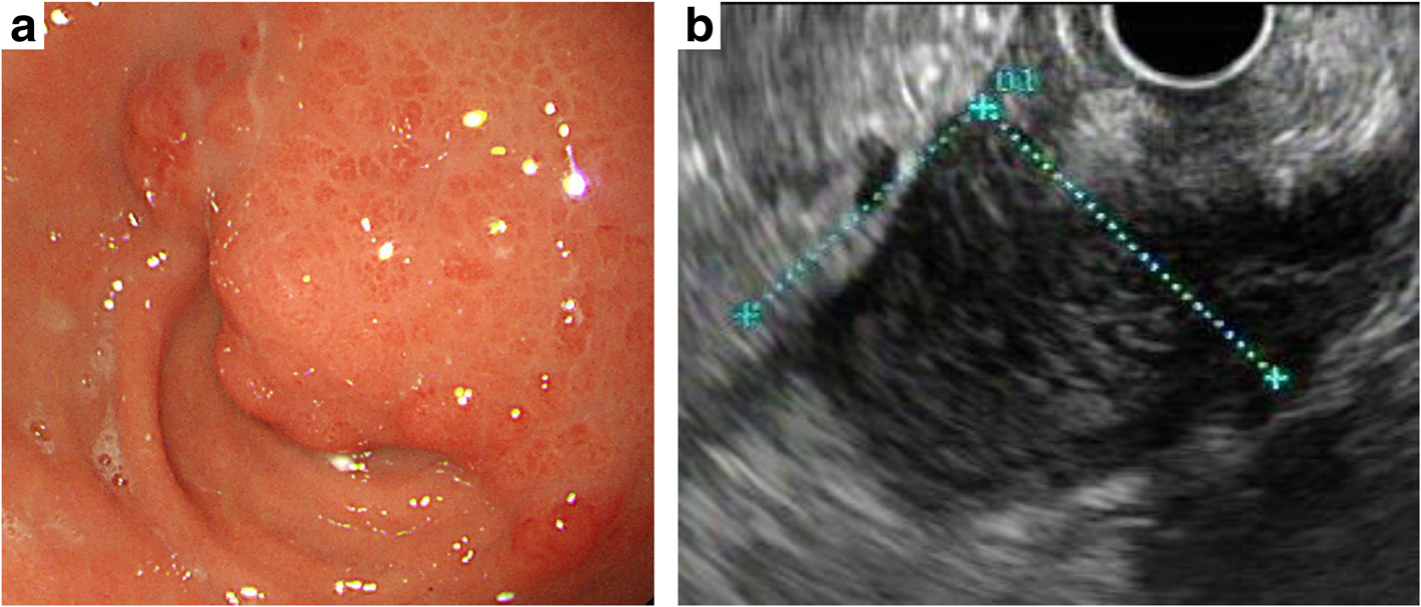
Endoscopic findings. **a** Upper endoscopy showing a submucosal tumor at the prepyloric antrum causing obstruction of the gastric outlet. **b** Endoscopic ultrasound showing a hypoechoic tumor located in the submucosal layer

**Fig. 3:**
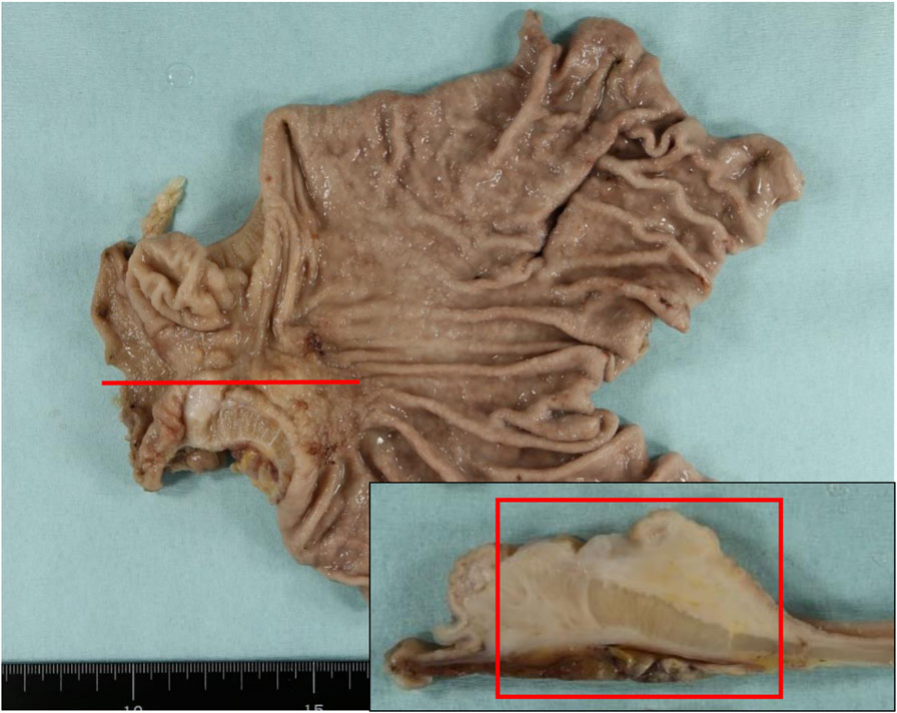
Macroscopic findings. The resected specimen revealed circumferential wall thickening of the prepyloric antrum (*red line*: cut line). Cut-section reveals thickening of the submucosal layer and the muscularis propria layer (*red square*: A cut surface of the resected specimen shown in Fig. 4a)

**Fig. 4:**
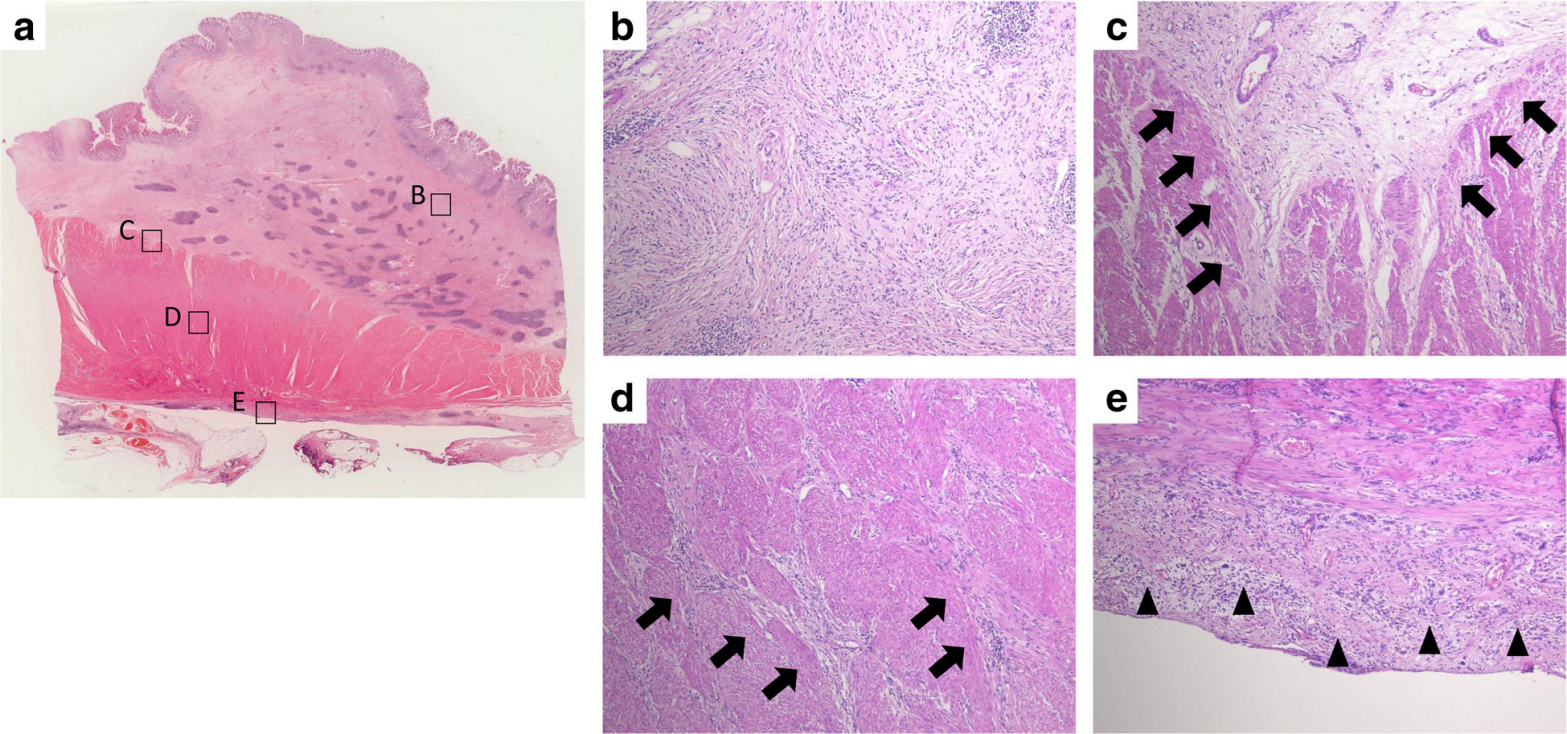
Microscopic findings. **a** Histopathological view through a low-power microscope. **b-e** Histopathological view through a medium-power microscope. Microscopic findings reveal spindle cell proliferation and infiltration of lymphocytes and plasma cells in the submucosal layer. Cells invading the muscularis propria layer (*arrows*) and extending to the subserosal layer (*arrowheads*)

**Fig. 5:**
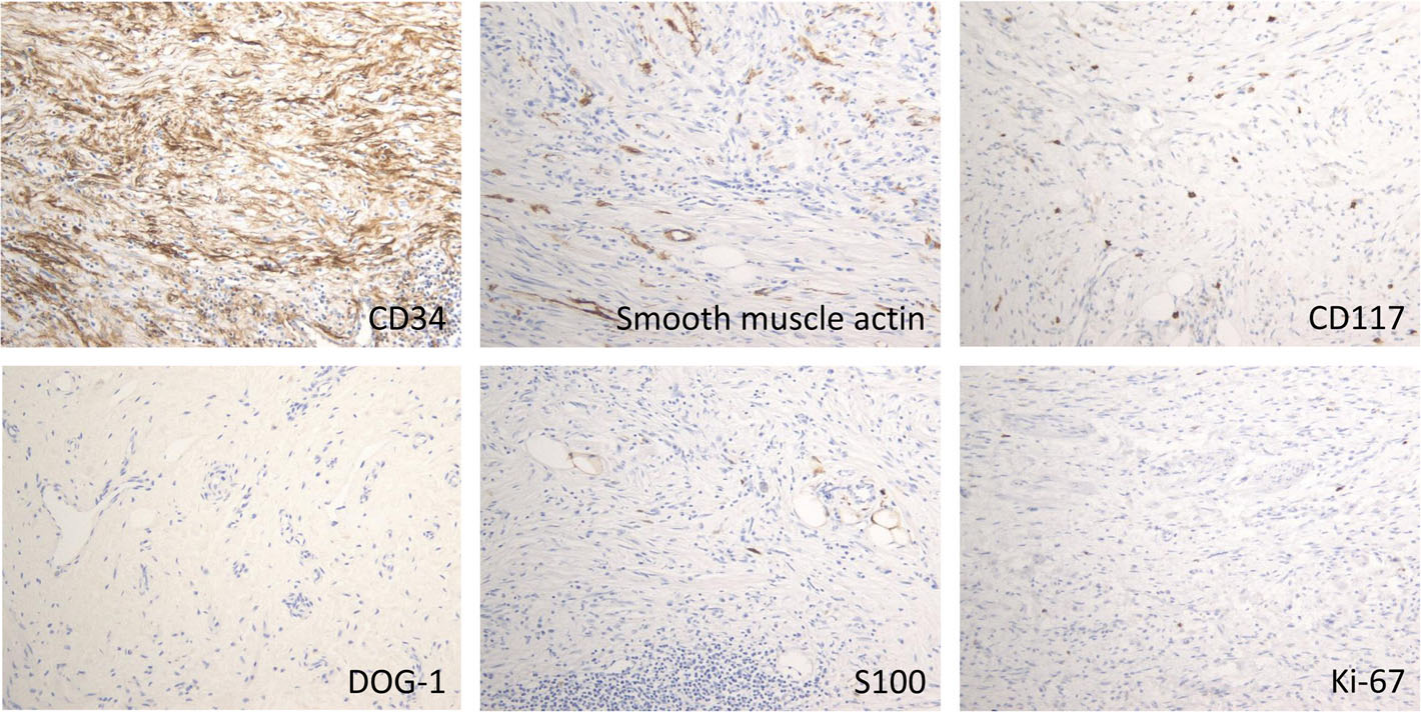
Immunohistochemical findings. Immunohistochemical findings revealing spindle cells stained positive for CD34 and smooth muscle actin but negative for CD117, DOG-1 and S100 protein. The Ki-67 labeling index was less than 1%. Scattered mast cells stained positive for CD117, but the spindle cells stained negative. Adipose tissue engulfed by the lesion stained positive for S100 protein, but the spindle cells stained negative

A 62-year-old woman presented with complaints of epigastric pain and vomiting for a few weeks. She had a history of traffic trauma followed by a brain abscess and had also undergone a total hysterectomy for a uterine myoma. She was given an antiepileptic agent, a non-steroidal anti-inflammatory drug and a proton pump inhibitor at a local hospital. She had no history of smoking or alcohol consumption. On physical examination, her blood pressure was 123/65 mmHg, her temperature 36.8 °C, her respiratory rate 18/min, and her heart rate 56 beats per minute. There was epigastric tenderness on palpation. Laboratory results indicated a white blood cell count of 7090/mm^3^ (67.5% neutrophils and 0.8% eosinophils), a hemoglobin level of 12.6 mg/dl, and a platelet count of 275,000/mm^3^. Other blood biochemistry data were normal. Serum anti-*Helicobacter pylori* (*H. pylori*) immunoglobulin G antibody was negative (4 IU/ml, < 10 cut-off). Computed tomography (CT) showed a 27-mm, hyper-enhancing tumor in the submucosa of the prepyloric antrum as well as thickening of the muscularis propria layer and a hyper-enhancing lesion in the subserosa (Fig. 1). Upper endoscopy also showed a submucosal tumor originating from the posterior wall of the prepyloric antrum (Fig. 2a). The tumor caused subtotal obstruction of the gastric outlet. Multiple biopsy specimens obtained from the surface of the submucosal tumor revealed infiltration of inflammatory cells. To obtain further histopathological information, endoscopic ultrasound-guided fine-needle aspiration was conducted. The endoscopic ultrasound showed a hypoechoic tumor with slightly heterogeneous internal echoes originating from the submucosal area (Fig. 2b). The tumor was punctured using a 22 G needle. The results indicated a small number of spindle cells, but a definitive diagnosis was not made. Considering her clinical findings, a diagnosis of GIST was suspected. Thus, distal gastrectomy was performed. The resected specimen revealed thickening of the submucosal layer and the muscularis propria layer at the antrum (Fig. 3). Histopathological examination revealed a submucosal tumor containing proliferating bland fibroblast-like spindle cells with inflammatory infiltrates (Fig. 4a and b). There was also a proliferation of small blood vessels. The inflammatory infiltrate contained lymphocytes and plasma cells. The spindle cells invaded the muscularis propria layer and extended to the subserosal layer (Fig. 4c, d and e). Regional lymph node metastasis was not detected. Immunohistochemical examination revealed that the spindle cells were diffusely positive for CD34, focally positive for alpha-smooth muscle actin and CD68, and negative for CD117, discovered on GIST-1 (DOG-1), S100 protein, desmin, cytokeratin AE1/AE3 and anaplastic lymphoma kinase (ALK). The Ki-67 (MIB-1) labeling index in the spindle cells was less than 1% (Fig. 5). Nuclear positivity for beta-catenin was not observed. IgG4-positive plasma cells were rare in the lesion. For mutational analysis, the tumor tissue was scraped from unstained slides, and DNA was extracted using a QIAamp DNA FFPE Tissue Kit (Qiagen, Hilden, Germany). The PDGFRA gene (exons 12, 14 and 18) was amplified by PCR using KOD FX Neo (Toyobo, Osaka, Japan). The PCR products were separated by electrophoresis and purified with a QIAquick Gel Extraction Kit (Qiagen). The purified PCR products were sequenced using an ABI 3130xl Genetic Analyzer (Applied Biosystems, Foster City, CA, USA). The results indicated the lack of mutations in the PDGFRA gene. While the invasive characteristics were atypical, the histopathological and immunohistochemical findings were consistent with an IFP. Thus, we finally diagnosed the tumor as an IFP. After resection, the patient’s clinical course was good: she has been closely followed up and has shown no recurrence.

## Discussion

IFPs were first described as eosinophilic submucosal granulomas by Vanek in 1949 [7], and after various names were suggested, Helwig and Ranier established the term IFP in 1953 [8]. IFPs can occur throughout the gastrointestinal tract, but they are most commonly found in the gastric antrum (66–75%) [9]. The growths are usually asymptomatic and incidentally found during endoscopic examinations performed for unrelated reasons. However, depending on the location and size of a lesion, various clinical presentations can occur. Similar to the present case, large gastric IFPs in the gastric antrum have been shown to cause subtotal obstruction of the gastric outlet [10]. Histologically, IFPs are submucosa-based lesions containing bland spindle cells with prominent vasculature. Their morphologic features often vary, and although IFPs are characterized by a perivascular “onion skin” appearance, approximately half of the lesions do not have this appearance [2]. Indeed, the present case did not have an “onion skin” appearance. In such cases, immunohistochemical examination is useful for diagnosing an IFP [11]. Immunohistochemically, IFPs are positive for CD34, smooth muscle actin and CD68 and negative for CD117, S100 protein and cytokeratin AE1/AE3 [2]. When histopathologically diagnosing IFPs, other spindle cell lesions, such as GISTs, schwannomas, and inflammatory myofibroblastic tumors (IMTs), must be ruled out, but they can be usually distinguished using immunohistochemical examination. For example, GISTs are positive for CD117, schwannomas are positive for S100 protein, and IMTs are positive for ALK and negative for CD34, whereas IFPs are not [2, 11–14]. In the present case, the most important differential diagnosis was that of an atypical GIST, because of CD117-negativity. However, because the present case was negative not only for CD117 but also for DOG-1, the possibility of the lesion being a GIST was less than 5% [15].

At least 1000 IFPs have been described thus far in the literature, and their etiology has been widely discussed [16]. Originally IFPs were hypothesized to result from robust host responses to an unknown local injury, infection, or allergic reaction [17]. Case reports describing morphological changes in IFPs after the eradication of *H. pylori* support this hypothesis [18]. However, because IFPs can also occur outside of the stomach, a causative role for *H. pylori* is difficult to ascertain. In 2008, Schildhaus et al. identified PDGFRA mutations in IFPs; these mutations had previously been detected only in GISTs [4]. The presence of PDGFRA mutations provides strong evidence of clonal proliferation and suggests that IFPs have a neoplastic nature. To date, IFPs are considered true neoplastic lesions rather than reactive lesions [5]. In 2012, Huss S et al. revealed that approximately 55% of IFPs have PDGFRA mutations and that most non-mutated IFPs are small lesions. To explain the existence of non-mutated IFPs exist, they proposed two hypotheses: (i) the low ratio of tumor cells in small lesions leads to false-negative results in mutational analysis; and (ii) mutational analysis can be negative because small lesions do not have oncogenic mutations and remain ‘pre-IFP’, but once the PDGFRA mutation occurs, the lesions start growing and evolving towards IFPs [19]. In the present case, despite the large size of the lesion, PDGFRA mutations were not detected. Thus, the mechanism by which some IFPs occur and grow in the absence of PDGFRA mutations remains unclear.

Although IFPs are generally considered benign, noninvasive lesions [1, 2], few cases have been observed to invade the muscularis propria layer [20]. With regard to gastric IFPs, only one invasive case was described in 2015 [21]. Here, we described a second case of an invasive gastric IFP. In the present case, spindle tumor cells invaded the muscularis propria layer and also proliferated in the subserosal layer. These findings imply that IFPs might occasionally behave as locally aggressive neoplasms with infiltrative growth patterns and may exhibit local recurrence after inadequate resection. Although endoscopic treatment is typically indicated for IFPs [22], when a lesion invades the muscularis propria layer, this treatment strategy can be inadequate. Indeed, a case of local recurrence after endoscopic removal has been reported [23]. In the present case, a hyper-enhancing lesion in the subserosa of the gastric antrum was detected on preoperative CT. Comparing CT findings with histopathological findings, the subserosal hyper-enhancing lesion shown on CT corresponded to the tumor invasion observed in the subserosal layer. For such cases, surgical treatment is recommended even with a preoperative diagnosis of IFP.

## Conclusion

In conclusion, we present a second case report of an invasive gastric IFP. Based on the present case, IFPs might be considered not only neoplastic but also potentially invasive lesions. If the invasive characteristics of IFPs are observed in further larger studies, the treatment strategy should be carefully considered to avoid inadequate treatment.

## Abbreviations

CT: Computed tomography
DOG-1: Discovered on gastrointestinal stromal tumor-1
GIST: Gastrointestinal stromal tumor
IFP: Inflammatory fibroid polyp
IMT: Inflammatory myofibroblastic tumor
PDGFRA: Platelet-derived growth factor receptor alpha

## References

[1] Stolte M, Finkenzeller G (1990). Inflammatory fibroid polyp of the stomach. Endoscopy.

[2] Liu TC, Lin MT, Montgomery EA, Singhi AD (2013). Inflammatory fibroid polyps of the gastrointestinal tract: spectrum of clinical, morphologic, and immunohistochemistry features. Am J Surg Pathol.

[3] Wille P, Borchard F (1998). Fibroid polyps of intestinal tract are inflammatory-reactive proliferations of CD34-positive perivascular cells. Histopathology.

[4] Schildhaus HU, Cavlar T, Binot E, Büttner R, Wardelmann E, Merkelbach-Bruse S (2008). Inflammatory fibroid polyps harbour mutations in the platelet-derived growth factor receptor alpha (PDGFRA) gene. J Pathol.

[5] Lasota J, Wang ZF, Sobin LH, Miettinen M (2009). Gain-of-function PDGFRA mutations, earlier reported in gastrointestinal stromal tumors, are common in small intestinal inflammatory fibroid polyps. A study of 60 cases. Mod Pathol.

[6] Heinrich MC, Corless CL, Duensing A, McGreevey L, Chen CJ, Joseph N, Singer S, Griffith DJ, Haley A, Town A, Demetri GD, Fletcher CD, Fletcher JA (2003). PDGFRA activating mutations in gastrointestinal stromal tumors. Science.

[7] Vanek J (1949). Gastric submucosal granuloma with eosinophilic infiltration. Am J Pathol.

[8] Helwig EB, Ranier A (1953). Inflammatory fibroid polyps of the stomach. Surg Gynecol Obstet.

[9] Akbulut S (2012). Intussusception due to inflammatory fibroid polyp: a case report and comprehensive literature review. World J Gastroenterol.

[10] Saritaş Ü, Üstündağ Y, Gedıkoğlu G (2011). Successful endoscopic treatment of huge gastric inflammatory fibroid polyp. Turk J Gastroenterol.

[11] Miettinen M, Sobin LH, Sarlomo-Rikala M (2000). Immunohistochemical spectrum of GISTs at different sites and their differential diagnosis with a reference to CD117 (KIT). Mod Pathol.

[12] Hirota S, Isozaki K, Moriyama Y, Hashimoto K, Nishida T, Ishiguro S, Kawano K, Hanada M, Kurata A, Takeda M, Muhammad Tunio G, Matsuzawa Y, Kanakura Y, Shinomura Y, Kitamura Y (1998). Gain-of-function mutations of c-kit in human gastrointestinal stromal tumors. Science.

[13] Sreevathsa MR, Pipara G (2015). Gastric schwannoma: a case report and review of literature. Indian J Surg Oncol.

[14] Makhlouf HR, Sobin LH (2002). Inflammatory myofibroblastic tumors (inflammatory pseudotumors) of the gastrointestinal tract: how closely are they related to inflammatory fibroid polyps?. Hum Pathol.

[15] Nishida T, Blay JY, Hirota S, Kitagawa Y, Kang YK (2016). The standard diagnosis, treatment, and follow-up of gastrointestinal stromal tumors based on guidelines. Gastric Cancer.

[16] Wysocki AP, Taylor G, Windsor JA (2007). Inflammatory fibroid polyps of the duodenum: a review of the literature. Dig Surg.

[17] Shimer GR, Helwig EB (1984). Inflammatory fibroid polyps of the intestine. Am J Clin Pathol.

[18] Hirasaki S, Matsubara M, Ikeda F, Taniguchi H, Suzuki S (2007). Gastric inflammatory fibroid polyp treated with helicobacter pylori eradication therapy. Intern Med.

[19] Huss S, Wardelmann E, Goltz D, Binot E, Hartmann W, Merkelbach-Bruse S, Büttner R, Schildhaus HU (2012). Activating PDGFRA mutations in inflammatory fibroid polyps occur in exons 12, 14 and 18 and are associated with tumour localization. Histopathology.

[20] Tajima S, Koda K. Locally infiltrative inflammatory fibroid polyp of the ileum: report of a case showing transmural proliferation. Gastroenterol Rep (Oxf). 2016; 10.1093/gastro/gow019. 10.1093/gastro/gow019 PMC5952929 27286722

[21] Lee JH, Yoo JS, Jung HY, Kim HM, Ryu H, Cho MY, Kim HS (2015). A case of invasion of Muscularis Propria of gastric inflammatory fibroid polyp. Korean J Helicobacter Up Gastrointest Res.

[22] Mavrogenis G, Herin M, Natale MD, Hassaini H (2016). Resection of a gastric fibroid inflammatory polyp by means of endoscopic submucosal dissection: how deep is deep enough?. Ann Gastroenterol.

[23] Zinkiewicz K, Zgodzinski W, Dabrowski A, Szumilo J, Cwik G, Wallner G (2004). Recurrent inflammatory fibroid polyp of cardia: a case report. World J Gastroenterol.

